# Clinical outcomes and outcome predictors of two-year assertive community treatment in Norway: an explorative prospective pre–post study

**DOI:** 10.1186/s12888-024-06181-5

**Published:** 2024-10-24

**Authors:** Torleif Ruud, Maria Lie Selle, Hanne K. Clausen, Kristin S. Heiervang, Sigrun Odden, Hanne Kilen Stuen, Anne Landheim

**Affiliations:** 1 Division of Mental Health Services, Akershus University Hospital, Lørenskog, Norway; 2 Institute of Clinical Medicine, University of Oslo, Oslo, Norway; 3 Health Services Research Unit, Akershus University Hospital, Lørenskog, Norway; 4 Norwegian National Advisory Unit on Concurrent Substance Misuse and Mental Health Disorders, Innlandet Hospital Trust, Brumunddal, Norway; 5 Faculty of Social and Health Sciences, Inland Norway University of Applied Sciences, Elverum, Norway

**Keywords:** Assertive community treatment, Severe mental illness, Outcomes, Predictors, Norway

## Abstract

**Background:**

Assertive Community Treatment (ACT) teams have become a part of mental health services for people with severe mental illness in many high-income countries. Studies in several countries have investigated the outcomes of ACT, and knowledge is also needed about outcomes of ACT teams in Norway. Our aims were to study clinical outcomes of ACT, how the outcomes were associated with characteristics of patients and treatment, and whether they differed across ACT teams.

**Methods:**

Our explorative, prospective, pre–post multicenter study involved 142 patients who received ACT for two years from the first 12 ACT teams established in urban and rural areas of Norway. There was no control group. The primary outcome was change in clinician-rated psychiatric symptoms. Secondary outcomes were clinician-rated change in functioning and engagement and change in community tenure compared with 2 years prior to ACT. We measured fidelity to the ACT model using the Tool for Measurement of Assertive Community Treatment. We performed linear mixed-effects modeling to analyze outcomes and their associations with characteristics of patients and treatment.

**Results:**

After two years, psychiatric symptoms were significantly reduced with a small effect size. Negative symptoms, anxiety and depression, and agitation and mania had significant reductions, while positive symptoms had nonsignificant changes. Functioning, engagement, and community tenure all significantly increased with small effect sizes. Age, difficulty to engage, problematic use of alcohol, frequent previous use of inpatient services, total number of sessions, and team’s fidelity to the ACT model were associated with different groups of symptoms. Less improvement in functioning was associated with team fidelity and number of sessions. Change in engagement was not associated with any predictors. Increased community tenure was greater for younger patients and patients who were on community treatment orders at treatment start.

**Conclusions:**

ACT for two years led to significant positive outcomes with small effect sizes for psychiatric symptoms, functioning, engagement, and community tenure. The outcomes were associated with some potential predictors, and some team-level variance emerged. Positive significant outcomes after two years indicate that larger improvements may be achieved from longer-term treatments by ACT teams.

**Supplementary Information:**

The online version contains supplementary material available at 10.1186/s12888-024-06181-5.

## Background

The assertive community treatment (ACT) model was developed 50 years ago to support people with severe mental illness to live in the community and reduce long-term inpatient stays in mental hospitals [1]. The early development of a scale for assessing ACT teams’ fidelity to the ACT model contributed to defining the model and to acknowledging ACT as an evidence-based practice [2–4]. Based on increasing experience and research, the ACT model has integrated new evidence-based practices and an emphasis on personal recovery, also leading to the development of an updated fidelity scale [5–8].

A Cochrane review in 2017 on intensive case management (ICM) including ACT and other types of ICM synthesized 40 randomized controlled trials (RCTs) involving 7,524 patients [9]. Although ACT was included as a type of ICM, no separate review on studies on ACT was conducted. In the 40 reviewed studies, reduced time in the hospital was the most frequently used outcome measure, and a meta-regression revealed that the degree of reduction was associated with how well ICM adhered to the ACT model. In their conclusion, the authors state, “We do think that the features of ICM that may improve outcome should be researched, as it may be that the model of intervention is effective only because of some of its features. This work may involve more observational studies in order to evolve the ICM model to new and better packages of care” [9].

The use of various instruments to measure clinical outcomes makes comparing clinical outcomes across studies somewhat difficult. In some ACT studies the Brief Psychiatric Rating Scale (BPRS) with 18 or 24 items or the Health of the Nation Outcome Scale has been used to measure the severity of psychiatric symptoms and problems, and the level of functioning has often been measured using the Global Assessment of Functioning Scale (GAF) [10–13].

The ACT model was developed to reach people with severe mental illness that were difficult to engage by the mental health services. This has been considered one of the key challenges to ACT teams and one of the key characteristics of the target group for ACT, and reduction in use of mental health inpatient services has been a common outcome measure on the success of ACT teams to engage and support the patients. This has also been measured as ‘community tenure’, which is the portion of days living in the community and not in mental health inpatient services [14]. However, it would also be useful to measure if the patients become more positive and engaged in their contact with the health services. A scale measuring this has been developed and tested by Park and colleagues [15].

Assessments of ACT teams’ fidelity to the ACT model have shed light on factors associated with positive outcomes of ACT. However, because fidelity is measured at the team level, it cannot capture any differences in service delivery to individual patients. Moreover, because the ACT model is a complex intervention involving numerous components, there is still limited knowledge about what are the most important ingredients and how they interact [7, 16]. There has also been increasing evidence that the reduction in use of inpatient beds shown in earlier ACT studies in the US is not found so much in more recent studies in European countries with lower use of inpatient beds and where various community mental health teams and services have integrated many aspects of the ACT model [17, 18] There is still a need for research on how clinical outcomes (e.g., psychiatric symptoms and patients’ functioning) are associated with patient characteristics and services provided to individual patients.

The first 12 ACT teams in Norway were established in 2009–2011 in urban and rural areas in all four of the country’s health regions. From the research-based evaluation of these teams, previous papers have been published on the fidelity of the ACT teams, the team members’ experiences with the ACT model, hospitalization compared for high and low users of inpatient service before and after ACT, and rehabilitation outcome compared for ACT users with and without substance use disorder [19–22].

### Aims

The aims of our study were to investigate (a) clinical outcomes of treatment by ACT teams in Norway, (b) associations between those outcomes and potential predictors (patients’ characteristics, number of ACT sessions, and ACT team’s fidelity to the ACT model), and (c) team differences in outcomes.

## Methods

### Context

In Norway, mental health services are mostly public services provided in 19 health trusts that also provide general hospital and other specialized health services [23]. Each health trust provides mental health and substance abuse services for adults at hospital inpatient units and two or more community mental health centers (CMHCs) serving local catchment areas. Each CMHC has outpatient clinics, mobile teams, and local inpatient units [24]. The CMHCs collaborate with general practitioners (GPs), primary health and social care in the municipalities, and hospital units, all sharing responsibility for the total mental health services. The municipalities and CMHCs collaborated on organizing and staffing the ACT teams established in 2009–2011 as required by the national health authorities for the ACTs to receive funding.

### Study design

Our study followed a prospective, explorative, pre–post design in evaluating the outcomes of ACT for two years using data from the national research-based evaluation of the first 12 ACT teams in Norway [25]. The study had no control group. In this article, we have followed version 4 of the Strengthening the Reporting of Observational Studies in Epidemiology (STROBE) statement for reporting cohort studies [26, 27].

### Sample

The sample consisted of 142 patients treated by the first 12 ACT teams in Norway, with a median of 13 patients per team (range 6–17). Patient characteristics at the start of treatment are shown in Table 1. Two-thirds were male, and mean age was 40 years, with an even distribution of age groups between the ages of 20 and 60 years. The most common diagnosis was schizophrenia spectrum disorder, followed by other severe disorders, bipolar disorder, and substance use disorders. The mean age at onset of those disorders was 26 years (SD = 9), and their mean duration was 13 years (SD = 9). Three-fifths had problematic use of alcohol or drugs at the treatment start. On average, patients had used inpatient mental health services for four months in the previous two years, and one-third were on community treatment orders (CTOs) at the start of the treatment. All patients were recruited for the study at the start of their ACT during the first year that the ACT team was in operation.

**Table 1:** Patient characteristics at the start of assertive community treatment (*N* = 142)

Variable	*N*	Mean (SD)	*n* (%)
**Age**	137	39.8 (10.6)	
20–29 years			27 (19.0)
30–39 years			44 (31.0)
40–49 years			34 (23.9)
50–59 years			29 (20.4)
≥ 60 years			3 (2.1)
Not registered			5 (3.5)
**Sex**	142		
Male (= 0)			95 (66.9)
Female (= 1)			47 (33.1)
**Psychosis**	142		
No (= 0)			35 (24.6)
Yes (= 1)			107 (75.4)
**Main diagnosis**	142		
Schizophrenia spectrum disorder			115 (81.0)
Bipolar disorder			9 (6.0)
Substance use disorder			4 (3%)
Other severe mental disorder			14 (10.0)
**Severity of psychiatric problems**			
BPRS total	137	2.46 (0.81)	
BPRS subscale Positive Symptoms	139	2.49 (1.27)	
BPRS subscale Negative Symptoms	139	2.49 (1.16)	
BPRS subscale Agitation and Mania	139	2.16 (1.09)	
BPRS subscale Anxiety and Depression	139	2.72 (1.01)	
**Functioning** (GAF-F)	139	39.6 (8.4)	
10–19			1 (0.7)
20–29			9 (6.3)
30–39			64 (45.1)
40–49			46 (32.4)
50–59			18 (12.7)
60–69			1 (0.7)
Not registered			3 (2.1)
**Engagement with health services** (HEAS)	139	9.67 (2.98)	
**Use of alcohol** (AUDIT)	142		
Not problematic use (= 0)			84 (59.2)
Problematic use (= 1)			58 (40.8)
**Use of drugs** (DUDIT)	142		
Not problematic use (= 0)			81 (57.0)
Problematic use (= 1)			61 (43.0)
**Community treatment orders** (CTO)	141		
Not CTO at start of ACT treatment (= 0)			90 (63.4)
CTO at start of ACT treatment (= 1)			51 (35.9)
Not registered			1 (0.7)
**Inpatient days in mental health units** (previous 2 years)	142	121 (155)	
**Community tenure** (of 730 d previous 2 years)	142	609 (155)	

The 12 ACT teams included 338 patients in their first year of operation, 178 (53%) of whom gave written informed consent to participate in the study. After two years, the teams completed the follow-up assessment of 142 of the patients. Of the other 36 patients, 16 had been discharged from the teams, five had died, and 12 had not received follow-up assessment. Compared to these 36 lost to follow-up assessment after two years as well as to all the 196 (58%) nonparticipants of the 338 patients included by the ACT teams in their first year of operation, the participants in our study had less severe psychiatric symptoms, better functioning, and fewer had problematic substance use. There were no differences in age, sex, diagnosis of severe mental illness, or being on community treatment order at the treatment start [20].

### Measures

#### Outcome variables

The primary outcome was change in psychiatric symptoms from treatment start to follow-up at 24 months (pre- value minus post- value, with positive change indicating improvement), measured using the BPRS-E [28, 29]. The rating scale and its instructions were translated to Norwegian by the first author (TR) in 1999 and used in a previous study in Norway [30]. Each of the 24 items are rated on a 7-point scale ranging from 1 (not present) to 7 (extremely severe). Based on factor analyses in several studies, the BPRS-E has four subscales: Positive Symptoms (grandiosity, suspiciousness, hallucinations, unusual thought content, bizarre behavior, disorientation, and conceptual disorganization), Negative Symptoms (blunted affect, emotional withdrawal, and motor retardation), Agitation and Mania (tension, uncooperativeness, excitement, distractibility, motor hyperactivity, and mannerism and posturing), and Anxiety and Depression (anxiety, depression, suicidality, and guilt) [31]. We estimated the interrater reliability (intraclass correlation coefficient [ICC]) of the BPRS-E and its subscales using ratings made by all the ACT teams regarding 20 anonymized cases treated and described by the various teams [32]. Following Koo and Li, we interpreted interrater reliability (ICC) to be poor (< 0.50), moderate (0.50–0.74), good (0.75–0.90), or excellent (> 0.90) [33]. Interrater reliability was moderate (0.54) for the total BPRS-E score, moderate for Positive Symptoms (0.71) and Agitation and Mania (0.72), poor for Negative Symptoms (0.44), and good (0.78) for Anxiety and Depression. Using the BPRS-E enabled us to compare our results with previous studies on ACT.

The three secondary outcome measures were changes in functioning, patients’ engagement, and community tenure. First, change in functioning from the treatment start to follow-up at 24 months (post- value minus pre- value, with positive change indicating improvement) was assessed by the ACT teams using the Function scale (GAF-F) of the split version of the Global Assessment of Functioning Scale (GAF), on which functioning and symptoms were scored separately [34, 35]. GAF-F scores range from 1 to 100, with higher scores indicating better functioning.

Second, the change in patients’ engagement from when treatment began to follow-up at 24 months (post- value minus pre- value, with positive change indicating improvement) was assessed by the ACT teams using the four-item version of the original five-item Homeless Engagement and Acceptance Scale (HEAS), as recommended by the developers when measuring the engagement of people who are not homeless [15]. The same strategy was previously employed in the Randomised Evaluation of Assertive Community Treatment in North London (REACT) study comparing ACT teams and community mental health teams (CMHTs) in the UK [36]. The four items, with response scales from 0 to either 3 or 4 with specific wordings for each step, were “How the client feels about you as a worker,” “The degree to which the client can be engaged,” “The client’s attitude to help,” and “The way the client engages with others.” Total scores for the HEAS range from 0 to 15, with higher scores indicating greater engagement. The scale has demonstrated good psychometric properties and predictive validity and is likely to be a useful tool in assessing engagement status [15]. The scale’s internal consistency in our study, measured in Cronbach’s alpha, was 0.78.

Last, community tenure is defined as the proportion of days living in the community instead of being inpatient in mental health services [14]. The change in community tenure was measured as the number of days in the community in the 2 years of ACT minus the days in the community in the two years before the start of the treatment.

#### Independent variables: patients’ characteristics and services provided

Several independent variables were included as potential predictors of outcomes, namely patients’ characteristics when treatment start, number of sessions with team members during the two years, and ACT team’s fidelity to the ACT model. Variables on patients’ characteristics at treatment start were age, sex, problematic use of alcohol, problematic use of drugs, and being on community treatment orders (CTO). We retrieved data on the use of inpatient mental health services in the 2 years before treatment start from the Norwegian Patient Registry.

Patients reported their use of alcohol on the Alcohol Use Disorder Identification Test (AUDIT) [37] and their use of drugs on the Drug Use Disorder Identification Test (DUDIT) [38]. AUDIT has 10 items, for a total score ranging from 0 to 40. DUDIT has 11 items, for a total score ranging from 0 to 44. On both questionnaires, higher scores indicate more severe problems. For both questionnaires, the total score was converted into a dichotomous variable of nonproblematic (0) or problematic use [1] based on the established cutoff point for each questionnaire (8 for men and 6 for women for AUDIT, 6 for men and 2 for women for DUDIT). A dichotomous variable for being on a community treatment order (CTO) or not at treatment start was registered as 1 or 0, respectively.

During the two-year follow-up, the ACT teams registered each session with each patient by completing a form including the date, location, and a code for the primary activity during the session. The total number of sessions was calculated from the form as a measure of the amount of contact and follow-up from the ACT team for each patient in the two-year period.

Each ACT team’s fidelity to the ACT model was assessed 12 and 30 months after the team’s establishment using the Tool for Measurement of Assertive Community Treatment fidelity scale (TMACT) [8]. TMACT has 47 items divided into six subscales: Operation and Structure, Core Team, Specialist Team, Core Practices, Evidence-Based Practices, and Person-Centered Planning and Practices. Fidelity was assessed by three groups of two researchers each who assessed four ACT teams, and each ACT team was assessed by the same group at 12 and 30 months. The assessments were performed according to the TMACT manual’s detailed guidelines and rules for data collection and rating after a weeklong training workshop by the developers of TMACT [39]. Each assessment was performed during a two-day onsite visit that involved interviewing team members, observing team meetings and team members’ home visits to patients, interviewing service users, reading the records of 10 randomly selected patients, and reviewing lists from the team on treatments provided to each patient. TMACT’s items were rated on a five-point scale from 1 (not implemented) to 5 (fully implemented) with definitions for five levels of fidelity. The fidelity scores for each subscale and the total scale were calculated as the mean score of the items. The fidelity ratings and comments were finalized after feedback from the team. We used the fidelity scores at 30 months in data analysis, because we considered those scores to be more representative of the follow-up period than scores at 12 months.

### Treatment provided by the ACT teams

The 12 first ACT teams in Norway were established between December 2009 and February 2011 mostly in smaller towns in rural areas but also in Norway’s largest cities. The number of team members on each team was between five and 12 full-time equivalents, and the patient-to-staff ratios were between three and 11. As required by the national health authority for the partial funding of the teams, all teams were established as a collaboration between the CMHC and the municipalities in catchment areas with populations from 40,000 to 100,000. The 12 teams registered 24,077 sessions with the 142 patients in our study during the two-year follow-up period, for a mean of 171 (SD = 104) sessions per patient, or 1.6 sessions per week on average. The primary activity coded for each session was “talking therapy” for 35% of the sessions, “medication management” for 17%, “practical support” for 9%, “social support or activity” for 7%, “substance use treatment” for 1%, and “not registered” for the remaining 31%.

The teams followed the ACT model meeting with patients in their homes or in the community, as 89% of the registered sessions were in these locations and only 10% in the team’s location. None of the teams operated 24 h/7 days a week, but some operated for a few hours in the evenings or in weekends. Mean fidelity scores ranging from 2.7 to 3.7 at 12 months and from 3.1 to 4.1 at 30 months indicated moderate to high fidelity. Thirty months after the teams were established, they showed a high-level implementation of the structural and organizational parts of the ACT model, whereas the recovery-focused and evidence-based treatments, as newer parts of the model, were implemented at a lower level. Scores on four of the six TMACT subscales improved from the first to second assessments [22].

### Data collection

Patients’ sociodemographic and clinical data were collected by the ACT teams at treatment start and at the two-year follow-up. Data at treatment start were collected from December 2009 to February 2012 at the recruitment of new patients during the first year after the teams were established, whereas data at the two-year follow-up were collected from December 2011 to February 2014. Data about psychiatric diagnoses, the severity of symptoms (BPRS-E), the level of functioning (GAF-F), and patients’ engagement (HEAS) were rated by ACT team members using all available information from observations and interviews with patients, as well as from their relatives, professionals in collaborating services, and their electronic medical records. The research group trained the ACT teams in using the patient assessment measurement tools in joint sessions organized for all ACT teams by the national health authority that funded the establishment of each team. Data on the frequency and severity of substance use (AUDIT and DUDIT) were collected using questionnaires that the patients completed alone or together with an ACT team member. Data on the patients’ use of inpatient mental health services during the 2 years with ACT and the 2 previous years were received from the Norwegian Patient Registry when these were available for all patients for all the 4 years.

### Data analysis

We reported descriptive statistics to describe the sample at treatment start, using SPSS for Windows version 28.

We used linear mixed effects models to assess changes in primary and secondary outcomes from the treatment start until the 2-year follow-up. The models contained fixed intercept and team to measure variance among teams. If team-level variance was negligible, then the model was reduced to a pairwise t-test or linear regression model. The results were presented as mean outcomes with 95% confidence intervals (CI) at treatment start, at follow-up, and for change. The estimated change from the linear mixed effects model was also presented with the corresponding p-value and as effect size (Cohen’s d) with 95% CI . We interpreted Cohen’s d of 0.20 as indicating a small effect, 0.50 as indicating a moderate effect, and 0.80 as indicating a large effect [40]. The intraclass correlation coefficient (ICC) was computed to show the proportion of variance at the team level.

The independent variables for data analyses of associations with the primary and secondary outcomes were selected based on which predictors we considered to be most important to include and the proportion of missing data for each. The regression analyses included approximately 130 informants, which limited the number of independent variables to 12.

To estimate the effect of possible predictors on changes in outcomes, the fixed effects in the linear mixed effects models were expanded to include preselected covariates. Stepwise backward reduction based on the Bayesian information criterion was used to reduce the number of fixed effects. If the number of fixed effects was reduced to fewer than five, then we stopped reducing the number of fixed effects to avoid underfitting the model.

The results were presented for both the full and final models as estimated regression coefficients with 95% CIs, p values, effect sizes, and coefficients of determination (R^2^) for the fixed part of the models. The effect sizes were Cohen’s d for all regression coefficients except “Previous inpatient days,” for which we estimated the coefficient for 30 days. The few patients with missing outcomes or covariates were excluded from the models, which left us with the same 128 patients in all models with predictors. We interpreted the minimum coefficient of determination (R^2^) to be small if 0.01, medium if 0.09, and large if 0.25 [40].

Model fit was evaluated by visual inspection of residual plots. For the model estimating outcome change in community tenure with predictors, the residuals showed slight heteroscedasticity. Due to this, we applied cluster-robust variance matrix estimators for the linear mixed models [41]. We used 5% significance level and did not correct for multiple testing. All models were fit using R version 4.3.0 (lmer/lm functions).

We used BPRS-E mean scores instead of sum scores in our data analyses and tables to facilitate the comparison of subscales with different numbers of items. However, to compare our results with other studies in the discussion we needed to use the BPRS-E sum score that had been reported by three other studies on ACT. We also needed to use community tenure per month in the discussion to compare our results with other studies reporting community tenure and use of inpatient services in days per month.

## Results

### Outcomes

Results for the primary and secondary outcomes appear in Table 2. The reduction in the primary outcome measure for severity of psychiatric symptoms (BPRS-E) after 2 years was significant and had a small effect size. Three of the BPRS-E subscales (Negative Symptoms, Anxiety and Depression, and Agitation and Mania) had a significant reduction in symptoms with a small effect size, while Positive Symptoms had nonsignificant changes. There were also positive significant outcomes with small effect sizes for all three secondary outcome measures, which indicated an increase in functioning (GAF-F), engagement (HEAS), and community tenure.

**Table 2:** Primary and secondary outcomes from 0 to 24 months estimated with linear mixed models

Outcome	*N*	Treatment start Mean (95% CI)	At 24 months Mean (95% CI)	Change Mean (95% CI)	*p*	Effect size: d * (95% CI)	ICC **
**Primary outcomes**							
BPRS total mean	137	2.46 (2.32, 2.59)	2.24 (2.11, 2.37)	0.20 (0.07, 0.33)	**0.03**	0.28 (0.05, 0.51)	0.08
BPRS Positive Symptoms	139	2.49 (2.27, 2.70)	2.39 (2.18, 2.60)	0.09 (-0.12, 0.29)	0.43	0.09 (-0.13, 0.31)	0.06
BPRS Negative Symptoms	139	2.49 (2.30, 2.69)	2.17 (2.01, 2.33)	0.31 (0.13, 0.50)	**0.02**	0.27 (0.08, 0.47)	0.03
BPRS Anxiety and Depression	139	2.72 (2.55, 2.89)	2.40 (2.24, 2.57)	0.33 (0.16, 0.49)	**0.01**	0.33 (0.12, 0.53)	0.05
BPRS Agitation and Mania	139	2.16 (1.98, 2.34)	1.94 (1.79, 2.09)	0.21 (0.05, 0.37)	**0.09**	0.20 (-0.01, 0.42)	0.06
**Secondary outcomes**							
GAF-F functioning	139	39.63 (38.23, 41.02)	42.60 (40.9, 44.29)	3.07 (1.48, 4.67)	**0.01**	0.33 (0.13, 0.54)	0.05
HEAS^***^	138	9.67 (9.17, 10.17)	10.96 (10.49, 11.42)	1.37 (0.72, 2.02)	**< 0.01**	0.35 (0.18, 0.52)	-
Community tenure	142	609 (584, 635)	669 (656, 681)	59 (33, 86)	**< 0.01**	0.37 (0.18, 0.56)	0.03

*) Effect size: Cohen’s d with 95% confidence intervals (CI)

**) Intraclass correlation coefficients (ICC) showing variance on team level

***) For HEAS, the variance between ACT teams was 0, and the model was reduced to a pairwise *t* test

### Predictors

Results of the final linear mixed models in regression analyses for associations between the primary outcomes and potential predictors appear in Table 3. Improvement in mean BRPS-E total score was significantly associated with increased age, not having a problematic use of alcohol, and being difficult to engage at treatment start. Associations with changes in BPRS-E subscales indicate which predictors were significantly associated with different groups of symptoms. Age, for instance, was associated with improved scores for Positive Symptoms, while being difficult to engage when treatment began was associated with improved scores for Negative Symptoms and Agitation and Mania. Not having a problematic use of alcohol was associated with improved scores for Negative Symptoms. Lower use of inpatient services before ACT was slightly associated with improved scores for Agitation and Mania. Fewer sessions with ACT team members were associated with improved scores for Negative Symptoms. Team’s fidelity to the ACT model was negatively associated with improved scores for Negative Symptoms. None of the predictors were significantly associated with changes in scores for Anxiety and Depression. The coefficients of determination (R^2^) revealed that the predictors in the final models explained 7–19% of the variation in changes in outcomes. Variation at team level ranged from 3 to 8%. The results of the full models for the associations between the primary outcomes and potential predictors appear in Table A in the Supplementary Material.

**Table 3:** Associations between primary outcomes and predictors for the final linear mixed models

Models and variables	RC Estimate (95% CI) *	*p*	Effect size: d **
**BPRS total scale**,** final model**, *R*^2^ = 0.18			
Intercept	-0.152 (-0.974, 0.670)	0.718	0.17
Age	0.015 (0.004, 0.025)	**0.007**	0.22
HEAS sum at treatment start	-0.066 (-0.104, -0.028)	**0.001**	-0.28
GAF-F at treatment start	0.012 (-0.002, 0.026)	0.103	0.14
AUDIT (above lim. = 1) at treatment start	-0.26 (-0.492, -0.028)	**0.030**	-0.36
Team-level variance *** 8%			
**BPRS Positive symptoms**,** final model**, *R*^2^ = 0.14			
Intercept	-1.199 (-2.61, 0.213)	0.099	0.16
Age	0.029 (0.011, 0.047)	**0.002**	0.26
HEAS sum at treatment start	-0.061 (-0.125, 0.004)	0.069	-0.15
GAF F at treatment start	0.022 (-0.003, 0.046)	0.089	0.15
AUDIT (above lim. = 1) at treatment start	-0.363 (-0.761, 0.036)	0.077	-0.30
Team-level variance *** 6%			
**BPRS Negative symptoms**,** final model**, *R*^2^ = 0.19			
Intercept	5.093 (2.353, 7.833)	< 0.001	0.18
HEAS sum at treatment start	-0.103 (-0.162, -0.044)	**0.001**	-0.28
AUDIT (above lim. = 1) at treatment start	-0.498 (-0.863, -0.133)	**0.009**	-0.45
Number of sessions	-0.002 (-0.004, -4e-05)	**0.015**	-0.22
Team fidelity at 30 months	-0.907 (-1.617, -0.197)	**0.014**	-0.23
Team-level variance *** 3%			
**BPRS Anxiety and Depression**,** final model**, *R*^2^ = 0.07			
Intercept	-0.276 (-1.482, 0.93)	0.655	0.13
Age	0.012 (-0.004, 0.028)	0.133	0.13
HEAS sum at treatment start	-0.041 (-0.097, 0.015)	0.153	-0.13
GAF F at treatment start	0.016 (-0.005, 0.037)	0.131	0.14
AUDIT (above lim. = 1) at treatment start	-0.286 (-0.629, 0.057)	0.105	-0.29
Team-level variance *** 5%			
**BPRS Agitation and Mania**,** final model**, *R*^2^ = 0.13			
Intercept	0.725 (0.186, 1.264)	0.010	-0.04
Sex (female = 1)	0.262 (-0.048, 0.571)	0.100	0.29
HEAS sum at treatment start	-0.065 (-0.115, -0.016)	**0.011**	-0.22
CTO (yes = 1) at treatment start	0.266 (-0.056, 0.588)	0.108	0.30
Inpatient days in previous 2 years	-0.001 (-0.002, -2e-04)	**0.015**	-0.04
Team-level variance *** 6%			

*) Estimated regression coefficients with 95% confidence intervals (CI)

**) Effect size: Cohen’s d with 95% confidence intervals (CI)

***) Intraclass correlation coefficient (ICC) calculated using empty linear mixed models with only intercepts

The results of the final linear mixed models in regression analyses for associations between the three secondary outcomes and potential predictors appear in Table 4. The improvement in functioning (GAF-F) was not significantly associated with any characteristic of patients at treatment start. However, it was significantly negatively associated with the number of sessions per patient and team’s fidelity, the two variables regarding the services provided by the ACT teams. None of the potential predictors were significantly associated with changes in patients’ engagement. For community tenure, younger patients showed better improvement than older ones, and patients who were on CTOs at treatment start showed better improvement than those not on CTOs. The coefficients of determination (R2) show that the predictors in the final models explained 5–14% of the variation in changes in outcomes. Variation at the team level ranged from 0 to 5%. Results of the full models for associations between the secondary outcomes and potential predictors appear in Table B in the Supplementary Material.

**Table 4:** Associations between secondary outcomes and predictors for the final linear mixed models

Models and variables	RC estimate (95% CI) *	*p*	Effect size: d **
**Functioning (GAF F)**,** final model**, *R*^2^ = 0.09			
Intercept	43.046 (14.403, 71.689)	0.009	0.03
Sex (female = 1)	2.001 (-1.441, 5.443)	0.257	0.21
BPRS mean at treatment start	-2.289 (-5.701, 1.123)	0.191	-0.24
Number of sessions	-0.019 (-0.037, -0.001)	**0.034**	-0.21
Team fidelity at 30 months	-10.193 (-17.743, -2.643)	**0.020**	-0.29
Team-level variance *** 5%			
**Engagement (HEAS) sum**,** final model**, *R*^2^ = 0.05			
Intercept	-0.157 (-3.309, 2.995)	0.922	0.11
Age	0.047 (-0.022, 0.116)	0.185	0.12
DUDIT (above lim. = 1) at treatment start	-0.765 (-2.237, 0.707)	0.310	-0.19
CTO (yes = 1) at treatment start	0.61 (-0.909, 2.129)	0.432	0.15
Inpatient days in previous 2 years	-0.003 (-0.007, 0.001)	0.262	-0.02
Team-level variance **** 0%			
**Community tenure**,** final model**, *R*^2^ = 0.14			
Intercept	128.429 (-3.263, 260.121)	0.094	-0.20
Age	-2.47 (-4.171, -0.769)	**0.021**	-0.16
BPRS mean at treatment start *****	-30.165 (-55.631, -4.699)	0.051	-0.14
GAF-F at treatment start	7.707 (-1.442, 16.856)	0.138	0.15
CTO (yes = 1) at treatment start	84.146 (31.026, 137.266)	**0.012**	0.52
Team-level variance *** 3%			

*) Estimated regression coefficients with 95% confidence intervals (CI)

**) Effect size: Cohen’s d with 95% confidence intervals (CI)

***) Intraclass correlation coefficient calculated using empty linear mixed models with only intercepts

****) Linear mixed model reduced to a linear regression model

*****) Non-correspondence between 95% CI and p value due to degrees of freedom based on number of groups

instead of number of individuals when applying cluster-robust variance matrix estimators

### Team differences in outcomes

Differences in outcomes across teams are also reported in the tables. ACT team was included as a second-level variable in the analyses of linear mixed-effects models both for the outcomes reported in Table 2 and in the regression analyses of associations between outcomes and potential predictors in Tables 3 and 4. ICCs in Table 2 show that 3–8% of the variance occurred among teams for all outcomes except patients’ engagement (HEAS) with no team-based differences. As reported in Tables 3 and 4, the variance at the team level for associations between outcomes and potential predictors was 8% for BPRS-E total and 3–6% for the subscales, 5% for functioning (GAF-F), 0% for patients’ engagement (HEAS), and 3% for community tenure.

## Discussion

Overall, psychiatric symptoms, functioning, patient engagement, and community tenure had significant improvements over two years. Improvement in symptoms was associated with age, not having a problematic use of alcohol, and being difficult to engage at treatment start. Less improvement in functioning was associated with number of sessions and the team’s fidelity to the ACT model. Increase in community tenure was greater for younger patients and patients being on CTOs at treatment start.

### Outcomes

Outcomes of our pre-post study may be compared to similar studies or to group differences in randomized controlled trials (RCTs). From the information in the 2017 Cochrane review, we identified three other studies on ACT that had used the BPRS-E as an outcome measure for psychiatric symptoms [9]. In our study, the mean BPRS-E total score was 59.0 at treatment start and 53.8 after 24 months (change of 5.2). In a US RCT with 223 patients with severe mental illness and co-occurring substance use disorders, the BPRS-E total scores at treatment start and 24 months were 44.8 and 42.5 (change of 2.3) for ACT and 46.9 and 42.3 (change of 4.6) for standard treatment, with improvement over time for both groups but a nonsignificant between-group difference in change [42]. By comparison, in a UK RCT with 251 patients, the BPRS-E total scores at treatment start and after 18 months were 36.4 and 32.9 (change of 3.5) for ACT and 36.2 and 33.5 (change of 2.7) for CMHTs, and a nonsignificant between-group difference in change [36]. Finally, in a Dutch RCT involving 118 patients, BPRS-E total scores at treatment start and after 12 months were 42 and 38 (change of 4.0) for ACT, and 45 and 42 (change of 3.0) for the control group with CMHTs, with significant improvement over time for both groups but a nonsignificant between-group difference [43]. None of those three RCTs revealed a significant between-group difference in change in psychiatric symptoms using the mean BPRS-E total scores. The severity of psychiatric symptoms measured using BPRS-E in our study exceeded those in the three above-cited studies, which may indicate that the patients in our study had more severe psychiatric symptoms. However, differences in how the rating scales were used may have influenced the results. Some differences may additionally be due to the use of different versions of the BPRS-E; although we used the same version 4 as in the UK and Dutch study, the researchers in the US study used version 3. The improvement in psychiatric symptoms in our study was significant but had a small effect size. However, the treatment of severe mental illness is often needed for several years, such that a significant though small effect size after two years may be promising and confirm ACT as an effective model of treatment and service delivery. Contrary to the three studies cited above, our study had no control group, and we cannot rule out that some of the change was due to regression toward the mean or other factors.

For functioning (GAF-F), we found significant improvement with a small to medium effect size. The mentioned Cochrane review on ICM reported five studies that had used the GAF and that GAF scores favored the experimental groups with ICM [9]. However, the review could not offer any conclusions on the improvement of functioning across studies on ACT at large, because the studies mostly used different instruments that measured different aspects and domains of functioning.

In our study, patients’ engagement score (HEAS) improved significantly from 9.67 to 10.96, for a change of 1.37, with a small effect size, as shown in Table 2. These results indicate that the patients had become more likely to use the treatment and support, for a potentially greater benefit over time. The RCT in UK using HEAS as a secondary outcome measure only measured HEAS at 18 months and could not report changes in HEAS over the 18 months [36]. However, they found a significant difference of 1.1 in HEAS score between ACT teams (score 9.1) and CMHTs (score 8.0) at 18 months, which is comparable to the pre–post change in our study. Our results thus confirm that HEAS is useful for measuring patients’ engagement.

Community tenure in our study was, on average, 59 days greater during the two years of ACT than during the two previous years, which is 2.5 days per month. These results may be compared to reports on the use of inpatient services or community tenure in many other studies. A meta-analysis in the Cochrane review from 2017 that included 24 RCTs with 3,595 patients showed that the number of days in the hospital per month was 0.86 days less for ICM than for standard care [9]. That rate is lower than the increase in community tenure in our study. In the US RCT mentioned above the mean increase in days in the community (community tenure) from baseline to two years was 0.2 days per month more for ACT than for individual case management, and with no significant between-group difference in change [42]. In the UK RCT mentioned above the reduction in hospital days (increase in community tenure) from baseline to 18 months was 1.0 days per month more for ACT than CMHC, which was a nonsignificant between-group difference in change [36]. Last, in the Dutch RCT, days in the hospital were 0.2 days more per month for ACT than for standard care during 15–24 months of treatment than for the previous year, with a nonsignificant between -group difference in change [43]. A previous article regarding the same 142 patients in our study reported different patterns in hospitalization for patients with high and low use of inpatient services [19]. High users demonstrated a greater reduction in hospital use during ACT (from more than 100 days per year on average before ACT to 35 days per year on average during ACT) while low users had an increase in hospitalization in the first year with ACT and a decrease in the second year (from a mean of 13 days per year before ACT to a mean of 29 and 21 days in the first and second years during ACT, respectively). A systematic review of RCTs on ICM involving meta-regression techniques showed that ICM can reduce hospital use when it is high, but is less successful when hospital use is already low [17]. The increase in community tenure in our study was equal to or higher than in the other studies cited above.

### Predictors

Associations between changes in outcomes and potential predictors have not been analyzed in most studies on ACT, which makes it difficult to compare such associations between other studies and ours.

Patients’ age was positively associated with improvement in psychiatric symptoms, especially positive symptoms, but negatively associated with community tenure. Sex, by contrast, was not significantly associated with any outcome. A diagnosis of psychosis (e.g., schizophrenia spectrum disorder or bipolar disorder) was not significantly associated with any outcome, either.

Improvement of functioning (GAF-F) was not significantly associated with any patient characteristics at treatment start. However, it was negatively associated with the number of ACT sessions and team’s fidelity to the ACT model, which are the two included variables on the services provided by the ACT teams. A negative association with the number of sessions may indicate that the team considered that patients with increased improvement in functioning needed less intensive follow-up. However, it is difficult to understand the negative association between patients’ improvement in functioning and the team’s fidelity of the ACT model. Additional post hoc regression analyses of the associations between GAF-F and the TMACT’s six fidelity subscales showed a significant negative association with the Core Team subscale measuring the functioning and capacity of the team leader, psychiatrist, and nurses on the ACT team, but no significant association to any of the other five subscales. It is difficult to determine whether this finding relates to differences between teams in factors we have not measured or is a random significant result perhaps related to small variance in fidelity across teams as commented below. To our knowledge, no other studies on ACT have analyzed associations between GAF and fidelity to the ACT model.

Improvement in patients’ engagement (HEAS) was not significantly associated with any potential predictor or the variables regarding services provided by the team. To our knowledge, no other study on ACT has examined the associations between patients’ engagement and potential predictors. However, an ongoing study of two hundred American ACT teams finds a negative correlation between high fidelity and the percent of patients who drop out of services, indicating that higher fidelity ACT teams may be more skillful in engaging and retaining more challenging patients (Personal communication 26 March 2024 from Lorna Moser, UNC School of Medicine).

Improvement in community tenure was negatively associated with age but positively associated with being on CTO. In earlier research on our sample, frequent hospital users were younger, more often lived alone, and more often were on CTOs than infrequent users [19]. Being on a CTO may have contributed to better follow-up and more stability in living in the community and managing without frequent use of hospital care.

Among other findings, a problematic use of alcohol (AUDIT) was negatively associated with improvement of both positive and negative symptoms but not significantly associated with any other outcomes. A problematic use of drugs (DUDIT), meanwhile, was negatively associated with patients’ engagement but not significantly associated with any other outcomes. These findings indicate that problematic substance abuse is a significant challenge in treatment of patients with severe mental illness, also for ACT teams.

### Team differences in outcomes

A small portion of the variance in changes in outcomes and in the associations with potential predictors indicate some differences between the 12 ACT teams, except for patients’ engagement (HEAS). Those findings align with the moderate variation in total fidelity (range = 3.1–4.1) found at the assessment of fidelity for the 12 teams at 30 months [22]. The fidelity of Norway’s ACT teams was slightly lower than reported for the 10 US ACT teams included in pilot-testing the TMACT [8]. The mean fidelity scores for TMACT total and subscales for the Norwegian ACT teams after 30 months operation were quite similar to the mean fidelity of more than two hundred American ACT teams in an ongoing study, except a lower fidelity for Norwegian teams on the Specialist Team subscale (Personal communication 26 March 2024 from Lorna Moser, UNC School of Medicine). However, the range of mean fidelity was smaller for the Norwegian teams, making it less possible to find significant differences and associations regarding fidelity.

### Strengths and limitations

One strength of our study was its use of comprehensive, well-designed measurement tools (BPRS-E, GAF-F, HEAS, AUDIT, DUDIT, and TMACT). Second, the fidelity assessors were trained in using TMACT by the scale’s developers and engaged in extensive discussions to achieve a joint understanding of the procedures and ratings using TMACT. Third, the estimated interrater reliability for ACT team members’ ratings of the primary outcome (BPRS-E) was moderate for the total scale and moderate or good for three of the four subscales. There are several limitations. The study had no control group, and we cannot rule out that regression toward the mean or other factors contributed to the outcomes. The potential sample bias with less severely ill patients may have given less variance in the sample and lower associations between outcome and potential predictors. The narrow range of fidelity ratings across teams may have limited the ability to detect the impact of fidelity. Small team sizes with low staff-to-client ratios may also have influenced the results. The data collection was conducted by the teams that also delivered the ACT treatment, and the assessments were performed unblinded. We did not measure the interrater reliability of fidelity ratings or ACT team members’ ratings on the GAF-F and HEAS. Having only 142 patients in the study limited the number of predictors that could be included in analyses. The ACT teams were newly established and had limited experience with the ACT model when they began recruiting patients for the study.

## Conclusions

ACT treatment for 2 years led to significant positive outcomes with small effect sizes for patients’ psychiatric symptoms, functioning, engagement, and community tenure. The outcomes were associated with some potential predictors, and some team-level variance indicates that some differences in outcomes occurred between teams. Positive significant outcomes after 2 years indicate that larger improvements may be achieved from longer-term treatment by ACT teams.

## Electronic supplementary material

Below is the link to the electronic supplementary material.

Supplementary Material 1

## Data Availability

The data set used and analyzed in the study is available from the corresponding author upon reasonable request.

## Abbreviations

ACT: Assertive community treatment
AUDIT: Alcohol Use Disorder Identification Test
BPRS: Brief Psychiatric Rating Scale
BPRS-E: Brief Psychiatric Rating Scale, Expanded
CI: Confidence interval
CMHC: Community mental health center
CMHT: Community mental health team
CTO: Community treatment order
DUDIT: Drug Use Disorder Identification Test
FACT: Flexible assertive community treatment
GAF: Global Assessment of Functioning Scale
GF: General practitioner
HEAS: Homeless Engagement and Acceptance Scale
ICC: Intraclass correlation coefficient
RCT: Randomized controlled trial
REACT: Randomised Evaluation of Assertive Community Treatment
TMACT: Tool for Measurement of Assertive Community Treatment
