# Supplementary material for: Clinical outcomes and outcome predictors of two-year assertive community treatment in Norway: an explorative prospective pre–post study
Source: BMC Psychiatry. 2024 Oct 24;24:729. doi: 10.1186/s12888-024-06181-5 (PMC11515545; doi:10.1186/s12888-024-06181-5)
Supplement: Supplementary file 1 — Supplementary Material 1 [file 12888_2024_6181_MOESM1_ESM.docx]

**Additional material to: Ruud T, Selle ML, Clausen H, Heiervang K, Odden S, Stuen HK, Landheim A. Clinical outcomes and outcome predictors of two-year assertive community treatment in Norway. An explorative prospective pre-post study. BMC Psychiatry (2024)**

**Table A: Estimated regression coefficients with 95% confidence intervals, p-values and effect sizes for the predictors of change in primary outcomes BPRS and subscales from 0 to 24 months, N = 128.**

| **Regression coefficient** | **Estimate (95% CI)** | **p value** | **Effect size** |
| --- | --- | --- | --- |
|  | **BPRS, full model**, R^2^ = 0.20 | | |
| Intercept | 1.043 (-1.724, 3.809) | 0.469 | 0.171 |
| Sex (female=1) | 0.165 (-0.087, 0.417) | 0.203 | 0.229 |
| Age | 0.010 (-0.002, 0.022) | 0.097 | 0.153 |
| Psychosis (yes=1) | 0.036 (-0.251, 0.323) | 0.806 | 0.050 |
| HEAS sum, 0 months | -0.063 (-0.102, -0.024) | **0.002** | -0.264 |
| GAF F, 0 months | 0.010 (-0.006, 0.025) | 0.225 | 0.109 |
| AUDIT (above lim. = 1) | -0.231 (-0.478, 0.016) | 0.069 | -0.321 |
| DUDIT (above lim. = 1) | -0.090 (-0.345, 0.164) | 0.487 | -0.125 |
| CTO (yes=1) | 0.037 (-0.224, 0.298) | 0.782 | 0.051 |
| Inpatient days previous 2 years | -0.001 (-0.001, 2e-04) | 0.149 | -0.025 |
| Number of sessions | -3e-05 (-0.001, 0.001) | 0.961 | -0.005 |
| Fidelity at 30 months | -0.262 (-0.962, 0.438) | 0.475 | -0.102 |
| Team level variance* 8% |  |  |  |
| **Regression coefficient** | **Estimate (95% CI)** | **p value** | **Effect size** |
|  | **BPRS positive, full model**, R^2^ = 0.15 | | |
| Intercept | 0.309 (-4.503, 5.121) | 0.901 | 0.097 |
| Sex (female=1) | 0.276 (-0.159, 0.71) | 0.216 | 0.229 |
| Age | 0.022 (0.001, 0.043) | **0.040** | 0.196 |
| Psychosis (yes=1) | 0.187 (-0.307, 0.681) | 0.459 | 0.156 |
| HEAS sum, 0 months | -0.058 (-0.125, 0.008) | 0.089 | -0.147 |
| GAF F, 0 months | 0.019 (-0.007, 0.046) | 0.150 | 0.134 |
| AUDIT (above lim. = 1) | -0.3 (-0.725, 0.125) | 0.170 | -0.250 |
| DUDIT (above lim. = 1) | -0.234 (-0.671, 0.204) | 0.298 | -0.194 |
| CTO (yes=1) | -0.075 (-0.524, 0.375) | 0.746 | -0.062 |
| Inpatient days previous 2 years | -0.001 (-0.002, 0.001) | 0.447 | -0.013 |
| Number of sessions | 0 (-0.002, 0.003) | 0.728 | 0.035 |
| Fidelity at 30 months | -0.368 (-1.589, 0.852) | 0.564 | -0.086 |
| Team level variance* 6% |  |  |  |
|  | **BPRS negative, full model**, R^2^ = 0.21 | | |
| Intercept | 5.354 (2.049, 8.659) | 0.002 | 0.275 |
| Sex (female=1) | 0.139 (-0.257, 0.535) | 0.491 | 0.126 |
| Age | -0.007 (-0.027, 0.013) | 0.473 | -0.069 |
| Psychosis (yes=1) | -0.227 (-0.690, 0.236) | 0.339 | -0.205 |
| HEAS sum, 0 months | -0.107 (-0.170, -0.044) | **0.001** | -0.295 |
| GAF F, 0 months | -0.001 (-0.025, 0.023) | 0.912 | -0.01 |
| AUDIT (above lim. = 1) | -0.457 (-0.847, -0.067) | **0.023** | -0.414 |
| DUDIT (above lim. = 1) | -0.149 (-0.553, 0.255) | 0.472 | -0.135 |
| CTO (yes=1) | 0.225 (-0.194, 0.644) | 0.294 | 0.204 |
| Inpatient days previous 2 years | 0 (-0.002, 0.002) | 0.800 | -0.004 |
| Number of sessions | -0.002 (-0.004, -4e-05) | **0.021** | -0.230 |
| Fidelity at 30 months | -0.841 (-1.611, -0.071) | **0.034** | -0.214 |
| Team level variance* 3% |  |  |  |
|  | **BPRS anxiety and depression, full model**, R^2^ = 0.09 | | |
| Intercept | -0.503 (-4.244, 3.239) | 0.795 | 0.247 |
| Sex (female=1) | -0.213 (-0.591, 0.165) | 0.271 | -0.216 |
| Age | 0.014 (-0.004, 0.033) | 0.124 | 0.155 |
| Psychosis (yes=1) | 0.066 (-0.367, 0.499) | 0.765 | 0.067 |
| HEAS sum, 0 months | -0.04 (-0.099, 0.018) | 0.179 | -0.123 |
| GAF F, 0 months | 0.019 (-0.004, 0.042) | 0.103 | 0.160 |
| AUDIT (above lim. = 1) | -0.282 (-0.652, 0.088) | 0.138 | -0.286 |
| DUDIT (above lim. = 1) | -0.038 (-0.42. 0.344) | 0.846 | -0.038 |
| CTO (yes=1) | -0.135 (-0.528. 0.258) | 0.502 | -0.137 |
| Inpatient days previous 2 years | 0 (-0.001. 0.001) | 0.755 | -0.006 |
| Number of sessions | 0.001 (-0.001. 0.003) | 0.471 | 0.077 |
| Fidelity at 30 months | 0.004 (-0.92. 0.928) | 0.993 | 0.001 |
| Team level variance* 5% |  |  |  |
|  | **BPRS agitation, full model**, R^2^= 0.14 | | |
| Intercept | 1.953 (-1.077, 4.984) | 0.221 | 0.080 |
| Sex (female=1) | 0.219 (-0.115, 0.552) | 0.202 | 0.244 |
| Age | 0.004 (-0.012, 0.021) | 0.603 | 0.051 |
| Psychosis (yes=1) | -0.139 (-0.525, 0.247) | 0.482 | -0.155 |
| HEAS sum, 0 months | -0.063 (-0.115, -0.011) | **0.019** | -0.213 |
| GAF F, 0 months | -0.004 (-0.023, 0.016) | 0.734 | -0.032 |
| AUDIT (above lim. = 1) | -0.14 (-0.468, 0.188) | 0.404 | -0.157 |
| DUDIT (above lim. = 1) | 0.141 (-0.198, 0.48) | 0.418 | 0.157 |
| CTO (yes=1) | 0.249 (-0.102, 0.599) | 0.167 | 0.277 |
| Inpatient days previous 2 years | -0.001 (-0.002, 0) | **0.037** | -0.038 |
| Number of sessions | -0.001 (-0.002, 0.001) | 0.447 | -0.079 |
| Fidelity at 30 months | -0.296 (-1.024, 0.432) | 0.441 | -0.093 |
| Team level variance* 6% |  |  |  |

*Intraclass correlation coefficient (ICC) calculated using empty linear mixed models with only intercepts.

**Supplementary material to: Ruud T, Selle ML, Clausen H, Heiervang K, Odden S, Stuen HK, Landheim A. Clinical outcome and outcome predictors of two years assertive treatment in Norwegian ACT teams. A prospective pre-post study. BMC Psychiatry (2024)**

**Table B: Estimated regression coefficients with 95% confidence intervals, p-values and effect sizes**

**for the predictors of change in secondary outcomes GAG-F, HEAS, and community tenure from**

**0 to 24 months, N = 128.**

|  | **GAF F, full model**, R^2^ = 0.11 | | |
| --- | --- | --- | --- |
| Intercept | 50.997 (18.322, 83.672) | 0.006 | 0.144 |
| Sex (female=1) | 1.620 (-2.065, 5.305) | 0.391 | 0.166 |
| Age | 0.076 (-0.108, 0.260) | 0.422 | 0.083 |
| Psychosis (yes=1) | -0.141 (-4.382, 4.100) | 0.948 | -0.014 |
| HEAS sum, 0 months | -0.365 (-0.955, 0.225) | 0.227 | -0.114 |
| BPRS mean, 0 months | -0.790 (-3.461, 1.881) | 0.563 | -0.062 |
| AUDIT (above lim. = 1) | -1.891 (-5.511, 1.729) | 0.308 | -0.194 |
| DUDIT (above lim. = 1) | -1.024 (-4.793, 2.745) | 0.595 | -0.105 |
| CTO (yes=1) | -2.019 (-5.886, 1.848) | 0.308 | -0.207 |
| Inpatient days previous 2 years | 0.002 (-0.010, 0.014) | 0.761 | 0.006 |
| Number of sessions | -0.015 (-0.035, 0.005) | 0.112 | -0.167 |
| Fidelity at 30 months | -11.621 (-19.820, -3.422) | **0.014** | -0.335 |
| Team level variance* 5% |  |  |  |
|  | **HEAS sum, full model**, R^2^ = 0.06 | | |
| Intercept | 0.213 (-13.703, 14.129) | 0.976 | 0.167 |
| Sex (female=1) | -0.194 (-1.760, 1.372) | 0.809 | -0.048 |
| Age | 0.041 (-0.037, 0.119) | 0.313 | 0.109 |
| Psychosis (yes=1) | -0.164 (-1.993, 1.665) | 0.861 | -0.041 |
| BPRS mean, 0 months | 0.483 (-0.652, 1.618) | 0.406 | 0.091 |
| GAF F, 0 months | 0.03 (-0.064, 0.124) | 0.542 | 0.061 |
| AUDIT (above lim. = 1) | -0.381 (-1.922, 1.160) | 0.629 | -0.095 |
| DUDIT (above lim. = 1) | -0.642 (-2.257, 0.973) | 0.438 | -0.16 |
| CTO (yes=1) | 0.533 (-1.123, 2.189) | 0.529 | 0.133 |
| Inpatient days previous 2 years | -0.002 (-0.008, 0.004) | 0.418 | -0.015 |
| Number of sessions | -0.003 (-0.011, 0.005) | 0.433 | -0.085 |
| Fidelity at 30 months | -0.469 (-3.583, 2.645) | 0.769 | -0.033 |
| Team level variance** 0% |  |  |  |
|  | **Community tenure, full model**, R^2^ = 0.16 | | |
| Intercept | 8.091 (-408.760, 424.942) | 0.971 | -0.405 |
| Sex (female=1) | 41.894 (-8.496, 92.284) | 0.140 | 0.261 |
| Age | -2.921 (-4.658, -1.184) | **0.011** | -0.194 |
| Psychosis (yes=1) | 11.211 (-32.360, 54.782) | 0.627 | 0.07 |
| BPRS mean, 0 months | -38.709 (-62.476, -14.942) | **0.015** | -0.183 |
| GAF-F, 0 months | 6.789 (-1.757, 15.335) | 0.158 | 0.128 |
| HEAS sum, 0 months | -1.196 (-3.74, 1.348) | 0.382 | -0.062 |
| AUDIT (above lim. = 1) | 33.542 (-30.634, 97.718) | 0.333 | 0.209 |
| DUDIT (above lim. = 1) | -8.981 (-59.921, 41.959) | 0.738 | -0.056 |
| CTO (yes=1) | 85.019 (30.457.,139.581) | **0.013** | 0.529 |
| Number of sessions | 0.103 (-0.156, 0.362) | 0.471 | 0.068 |
| Fidelity at 30 months | 46.017 (-25.668, 117.702) | 0.267 | 0.080 |
| Team level variance* 3% |  |  |  |

*Intraclass correlation coefficient (ICC) calculated using empty linear mixed models with only intercepts.

** Linear mixed model was reduced to linear regression model
